# Structural and mechanistic analysis of ATPase inhibitors targeting mycobacterial DNA gyrase

**DOI:** 10.1093/jac/dkaa286

**Published:** 2020-07-30

**Authors:** Sara R Henderson, Clare E M Stevenson, Brandon Malone, Yelyzaveta Zholnerovych, Lesley A Mitchenall, Mark Pichowicz, David H McGarry, Ian R Cooper, Cedric Charrier, Anne-Marie Salisbury, David M Lawson, Anthony Maxwell

**Affiliations:** d1 Department of Biological Chemistry, John Innes Centre, Norwich Research Park, Norwich NR4 7UH, UK; d2 Redx Pharma PLC, Mereside, Alderley Park, Alderley Edge SK10 4TG, UK; d11 Norwich Medical School, University of East Anglia, Norwich NR4 7UQ, UK; d12 Laboratory of Molecular Biophysics, The Rockefeller University, 1230 York Avenue, New York, NY 10065, USA; d13 Sygnature Discovery, The Discovery Building, Biocity, Pennyfoot Street, Nottingham NG1 1GR, UK; d14 Globachem Discovery Ltd, Mereside, Alderley Park SK10 4TG, UK; d15 AMR Centre Ltd, Mereside, Alderley Park SK10 4TG, UK; d16 IHMA Europe Sàrl, Rte. de I’lle-au-Bois 1A, 1870 Monthey/VS, Switzerland; d17 5D Health Protection Group Ltd, William Henry Duncan Building, West Derby Street, Liverpool L7 8TX, UK

## Abstract

**Objectives:**

To evaluate the efficacy of two novel compounds against mycobacteria and determine the molecular basis of their action on DNA gyrase using structural and mechanistic approaches.

**Methods:**

Redx03863 and Redx04739 were tested in antibacterial assays, and also against their target, DNA gyrase, using DNA supercoiling and ATPase assays. X-ray crystallography was used to determine the structure of the gyrase B protein ATPase sub-domain from *Mycobacterium smegmatis* complexed with the aminocoumarin drug novobiocin, and structures of the same domain from *Mycobacterium thermoresistibile* complexed with novobiocin, and also with Redx03863.

**Results:**

Both compounds, Redx03863 and Redx04739, were active against selected Gram-positive and Gram-negative species, with Redx03863 being the more potent, and Redx04739 showing selectivity against *M. smegmatis*. Both compounds were potent inhibitors of the supercoiling and ATPase reactions of DNA gyrase, but did not appreciably affect the ATP-independent relaxation reaction. The structure of Redx03863 bound to the gyrase B protein ATPase sub-domain from *M. thermoresistibile* shows that it binds at a site adjacent to the ATP- and novobiocin-binding sites. We found that most of the mutations that we made in the Redx03863-binding pocket, based on the structure, rendered gyrase inactive.

**Conclusions:**

Redx03863 and Redx04739 inhibit gyrase by preventing the binding of ATP. The fact that the Redx03863-binding pocket is distinct from that of novobiocin, coupled with the lack of activity of resistant mutants, suggests that such compounds could have potential to be further exploited as antibiotics.

## Introduction

Although TB, caused by the bacterium *Mycobacterium tuberculosis*, is often thought of as a disease of the past, in recent years there have been increasing rates of the disease. This is in part due to rising resistance to the current primary treatment regimen—directly observed treatment, short-course (DOTS)—and decreasing efficacy of the BCG vaccine (https://www.who.int/tb/publications/global_report/en/).^1^ To overcome this issue, there have been significant efforts to develop new treatments, which have, for the most part, been unsuccessful, the current first-line treatments having been used for over 50 years. One strategy is to find new compounds that exploit existing well-validated targets, such as DNA gyrase, which is a member of the DNA topoisomerase family of enzymes.^2^^,^^3^

DNA topoisomerases are enzymes that regulate the topology of DNA in all cells.^4^ They are classified into two types, I and II, depending on whether their reactions involve single (I)- or double (II)-stranded breaks in DNA. Gyrase is a type II topoisomerase and the only enzyme that can catalyse the introduction of negative supercoils into DNA;^3^^,^^5^ it is composed of two subunits, GyrA and GyrB, which form an A_2_B_2_ complex in the active enzyme. Gyrase is an essential enzyme in bacteria and has been extensively studied as a target for antibacterial agents;^6^ gyrase has been described as an effective drug target for the development of new anti-TB agents.^2^ Gyrase is the only type II topoisomerase in *M. tuberculosis* and, in addition to supercoiling, carries out functions that would be carried out by DNA topoisomerase IV (topo IV) in other bacteria, such as decatenation.^7^^,^^8^

Several small-molecule antibiotics and bacterial toxins target gyrase, the most prominent of which are the fluoroquinolones (FQs).^9^^,^^10^ Indeed, these compounds (e.g. moxifloxacin) have been utilized in TB therapy.^11^ FQs inhibit gyrase by stabilizing the DNA-cleavage complex, a transient intermediate in the DNA supercoiling cycle, which, *in vivo*, can lead to chromosome breaks and cell death.^9^^,^^10^ Other compounds, such as the aminocoumarins (e.g. novobiocin), inhibit gyrase through targeting the ATPase domains of gyrase and topo IV, located in the N-terminal domain of GyrB (and ParE), thus preventing supercoiling.^3^^,^^12^ Although aminocoumarins have not enjoyed the same clinical success as FQs, inhibition of the ATPase activity is a very effective way of inhibiting the enzyme, and the existence of several crystal structures of this region has enabled the development of a range of inhibitory molecules.^13^^,^^14^

Previously, compounds with a pyrrolopyrimidine core have been described as having broad-spectrum antibacterial activity through targeting the ATPase domains of gyrase and topo IV with high potency against Gram-positive bacteria and weaker activity against Gram-negative bacteria.^15^^,^^16^ The introduction of a third cyclic ring to the pyrrolopyrimidine core creates a tricyclic ring system with improved antibacterial efficiency against Gram-positive bacteria.^17^ The mechanism of action was determined to be due to inhibition of the ATPase activity of GyrB and ParE (the equivalent subunit of topo IV). Crystal structures of several of the compounds in the series, bound to a variety of Gram-negative GyrB and ParE subunits, have been solved by X-ray crystallography.^15–17^ The binding pocket inferred from crystallography shows that the pyrrolopyrimidine-containing compounds bind in a similar pocket to novobiocin in GyrB, but Arg136 (*Escherichia coli* numbering) does not make direct contact with the compounds, as distinct from novobiocin. More recently, these compounds have been further developed to create the current series with increased specificity against mycobacteria.^18^ Additionally, it has been shown that these compounds inhibit the supercoiling and ATPase reactions of *M. tuberculosis* gyrase without inhibiting human topoisomerase II, and maintain activity against a panel of relevant *M. tuberculosis* clinical isolates resistant to other commonly used drugs.^18^

**Figure 1.**
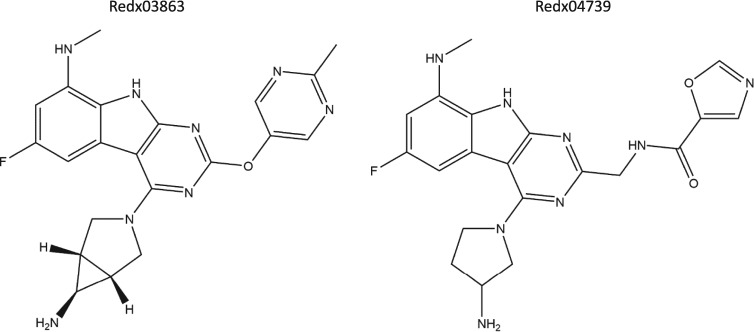
Structures of Redx03863 and Redx04739.

In this paper we have extended this work using two compounds (Figure 1) and show that they are effective inhibitors of *M. tuberculosis* gyrase and inhibit the growth of *Mycobacterium smegmatis*. In addition, we present crystal structures of the GyrB N-terminal ATPase sub-domain from *Mycobacterium thermoresistibile* bound to one of these compounds (Redx03863) and also to novobiocin, demonstrating that this novel compound class inhibits via a binding pocket and resistance mechanism different from that of novobiocin. It is important to note that all the residues that line the ATP pocket are conserved across *M. tuberculosis*, *M. smegmatis* and *M. thermoresistibile*, i.e. the latter two are good models for the former in this context.

## Materials and methods

### Antibacterial testing

Antibacterial testing was performed for *Acinetobacter baumannii* NCTC 13420, *E. coli* ATCC 25922, *M. smegmatis* ATCC 19420, *Pseudomonas aeruginosa* ATCC 27853 and *Staphylococcus aureus* ATCC 29213, using the broth microdilution method under CLSI guidelines^19^ (Table 1). The agar dilution method was additionally performed for *M. smegmatis* under EUCAST guidelines.^20^

**Table 1.** MIC values of Redx03863 and Redx04739 for selected bacterial strains

Bacterial strain	MIC (mg/L)	
	Redx03863	Redx04739
*A. baumannii* NCTC 13420	0.031	64
*E. coli* ATCC 25922	0.016	8
*M. smegmatis* ATCC 19420	0.016	0.25
*P. aeruginosa* ATCC 27853	4	32
*S. aureus* ATCC 29213	0.125	8

### Bacterial mutagenesis

Single colonies of *M. smegmatis* ATCC 19420 were grown for 48 h at 37°C with shaking in Middlebrook 7H9 broth supplemented with 10% (v/v) OADC, 0.2% (v/v) glycerol and 0.05% (v/v) Tween 80. Fifty microlitres of starter culture was used to inoculate 10 mL agar plates containing 4× agar MIC of Redx03863. Colonies were selected after 5 days of incubation at 37°C and confirmed by testing broth MIC values. Cross-resistance was tested for a panel of compounds, and the genomic DNA was extracted (EdgeBio PurElute™ Bacterial Genomic Kit, with bead beating lysis step). The DNA gyrase genes were amplified using PCR, purified (Qiagen PCR clean-up kit) and products sequenced (Eurofins Genomics) (Table S1, available as Supplementary data at *JAC* Online).

### Protein purification and crystallization

The *M. smegmatis* GyrB 22 kDa ATPase sub-domain was cloned, purified and crystallized as described,^21^ resulting in a double loop-deletion mutant of the 27 kDa N-terminal ATPase sub-domain. Crystals grown, using sitting-drop vapour diffusion, with 17 mg/mL protein in the presence of 1 mM novobiocin against 15% (w/v) PEG8000, 100 mM sodium acetate pH 5.6, 200 mM calcium acetate, were harvested in the precipitant supplemented with 25% (v/v) ethylene glycol for cryoprotection.

The *M. thermoresistibile* GyrB 21 kDa ATPase sub-domain (residues 20–213) was cloned to be analogous to the *M. smegmatis* construct with two potentially flexible loops (residues 103–123 and 214–244) removed. Purification was performed under the same conditions as for the *M. smegmatis* protein. Purified protein, at a concentration of 22 mg/mL, was supplemented with 1 mM novobiocin or 0.24 mM Redx03863, before crystallization by sitting-drop vapour diffusion against 100 mM zinc chloride, 100 mM ammonium acetate, 3.5%–5% (w/v) PEG6000, 3.5%–5% (w/v) PEG8000, 3.5%–5% (w/v) PEG1000 and 100 mM Bis-Tris pH 7.0–7.3 with assistance of seeding. Briefly, crystals containing novobiocin were harvested after 7–10 days in 40–100 μL of mother liquor, and vortexed vigorously for 4 min with three 1 mm glass beads in 1.5 mL Eppendorf tubes, and 0.1 μL was used to supplement future crystal trials with 0.2 μL screen and 0.3 μL of protein solution. Crystals were harvested in precipitant supplemented with 25% (v/v) ethylene glycol.

### X-ray data collection and structure solution

Data collection was performed on beamlines I03 and I04-1 at Diamond Light Source (Oxford, UK) with the crystals maintained at −173°C using a Cryojet cryocooler (Oxford Instruments). X-ray data were collected for 3600 × 0.1° images at a wavelength of 0.97 Å to a maximum resolution of 1.3 Å with a Pilatus3 6 M Hybrid Photon Counting detector (Dectris), then integrated and scaled using DIALS,^22^ via the XIA2 expert system,^23^ and merged using AIMLESS;^24^ the resulting data-collection statistics are summarized in Table 2. The *M. smegmatis* GyrB22-novobiocin complex structure was solved by molecular replacement with PHASER,^25^ using chain A from the published *M. smegmatis* structure (PDB code 4B6C)^21^ as a template. This was then used as a template to solve the *M. thermoresistibile* GyrB21-novobiocin complex structure, where the sequence identity between these ATPase domains is ∼80%. The molecular replacement models were rebuilt using BUCCANEER^26^ before being completed through iterative rounds of refinement (REFMAC^27^) and manual model building (COOT^28^). The initial model of the *M. thermoresistibile* GyrB21–Redx03863 complex structure was subsequently obtained by direct refinement of the protein component of the GyrB21–novobiocin complex, then completed as above. Structures were validated through MOLPROBITY^29^, PDB-REDO^30^ and the PDB-validation server.^31^ The refinement and validation statistics are summarized in Table 2. Omit *m*F_obs_ − *D*F_calc_ difference electron density for bound ligands was calculated using phases from the final models without the ligand after the application of small random shifts to the atomic coordinates, re-setting temperature factors and re-refining to convergence. All structural figures were prepared using CCP4mg.^32^

**Table 2.** Summary of X-ray data and model parameters for GyrB N-terminal sub-domains

	Protein		
	GyrB21	GyrB21	GyrB24
Organism	*M. thermoresistibile*	*M. thermoresistibile*	*M. smegmatis*
Drug	novobiocin	Redx03863	novobiocin
Data collection			
Diamond Light Source beamline	I03	I03	I04-1
wavelength (Å)	0.9700	0.9700	0.9159
detector	Pilatus 6M	Pilatus 6M	Pilatus 6M
resolution range (Å)	81.86–1.40 (1.42–1.40)	51.67–1.50 (1.53–1.50)	78.48–1.60 (1.63–1.60)
space group	*P*2_1_	*P*2_1_	C2
cell parameters (Å/°)	*a* = 43.96, *b* = 51.11, *c* = 83.21, β= 100.33	*a* = 43.77, *b* = 51.67, *c* = 82.91, β= 100.25	*a* = 156.96, *b* = 56.11, *c* = 50.69, β= 90.66
total no. of measured intensities	463106 (21837)	374604 (18170)	387422 (19275)
unique reflections	71663 (3548)	58503 (2863)	58273 (2853)
multiplicity	6.5 (6.2)	6.4 (6.3)	6.6 (6.8)
mean *I*/σ(*I*)	15.7 (1.7)	12.5 (1.2)	10.4 (2.0)
completeness (%)	100.0 (100.0)	100.0 (99.9)	100.0 (100.0)
* R*_merge_ ^a^	0.048 (0.875)	0.062 (1.455)	0.110 (1.071)
* R*_meas_ ^b^	0.052 (0.956)	0.067 (1.588)	0.119 (1.159)
* CC*_½_ ^c^	0.999 (0.849)	0.999 (0.679)	0.999 (0.647)
Wilson *B* value (Å^2^)	16.87	20.3	15.73
Refinement			
resolution range (Å)	81.86–1.40 (1.44–1.40)	43.69–1.50 (1.54–1.50)	78.48–1.60 (1.64–1.60)
reflections: working/free^d^	67991/3649 (4988/280)	55659/2826 (4079/208)	55300/2973 (4085/214)
* R*_work_/*R*_free_^e^	0.140/0.181 (0.240/0.257)	0.149/0.203 (0.278/0.320)	0.137/0.188 (0.260/0.310)
Ramachandran plot: favoured/allowed/ disallowed^f^ (%)	97.42/2.58/0.00	96.82/3.18/0.00	98.07/1.93/0.00
RMSD, bond distance (Å)	0.009	0.011	0.011
RMSD, bond angle (º)	1.513	1.659	1.514
No. of protein residues (ranges)	A: 20–102, 123–213, 245–254	A: 20–102, 123–213, 245–253	A: 16–102, 123–213, 246–255
	B: 22–88, 92–102, 123–213, 245–254	B: 22–87, 92–102, 123–213, 245–253	B: 18–102, 123–213, 246–255
no. of water/drug/zinc/ Na/EDO/act/PEG molecules	296/2/6/2/0/0/0	291/2/6/2/0/0/0	379/2/0/5/10/3
mean *B* factors: protein/ ions/ligands/waters (Å^2^)	23.4/25.5/26.8/37.8	26.7/31.5/26.5/38.0	22.5/29.0/26.7/35.0
RSCC scores for main ligand^g^	A: 0.96; B: 0.94 (for both conformers)	A: 0.94; B: 0.94	A: 0.96; B: 0.97
PDB accession code	6Y8L	6Y8N	6Y8O

Values in parentheses are for the outer resolution shell.

a *R*_merge_ = ∑_*hkl*_ ∑_*i*_ |*I_i_*(*hkl*) − ⟨*I*(*hkl*)⟩|/∑_*hkl*_ ∑*_i_I_i_*(*hkl*).

b *R*_meas_ = ∑_*hkl*_ [*N*/(*N* − 1)]^1/2^ × ∑_*i*_ |*I_i_*(*hkl*) − ⟨*I*(*hkl*)⟩|/∑_*hkl*_ ∑*_i_I_i_*(*hkl*), where *I_i_*(*hkl*) is the *i*th observation of reflection *hkl*, ⟨*I*(*hkl*)⟩ is the weighted average intensity for all observations *i* of reflection *hkl* and *N* is the number of observations of reflection *hkl*.

c *CC*_½_ is the correlation coefficient between symmetry equivalent intensities from random halves of the dataset.

d The dataset was split into ‘working’ and ‘free’ sets consisting of 95% and 5% of the data, respectively. The free set was not used for refinement.

e The *R*-factors *R*_work_ and *R*_free_ are calculated as follows: *R* = ∑(| *F*_obs_ − *F*_calc_ |)/∑| *F*_obs_ |, where *F*_obs_ and *F*_calc_ are the observed and calculated structure factor amplitudes, respectively.

f As calculated using MolProbity.

g Real space correlation coefficients from PDB validation server.

### Enzymes and assays

DNA gyrase from *M. tuberculosis* was purified as previously described.^33^ Supercoiling assays were carried out as described;^33^ ATPase assays were performed using the PK/LDH-linked assay.^34^ Gyrase from *M. thermoresistibile* was cloned and purified using methods analogous to those used for *M. tuberculosis* (see Supplementary data).

## Results and discussion

### Antibacterial activity

We initially tested the two compounds (Redx03863 and Redx04739) of interest against selected bacterial strains, including *E. coli* and *M. smegmatis*, to determine if they were selective for mycobacteria (Table 1). We found that Redx04739 showed a significantly lower MIC against *M. smegmatis* compared with the other species tested. Redx03863 was more potent and showed good activity against all five strains.

### Inhibition of DNA gyrase

**Figure 2.**
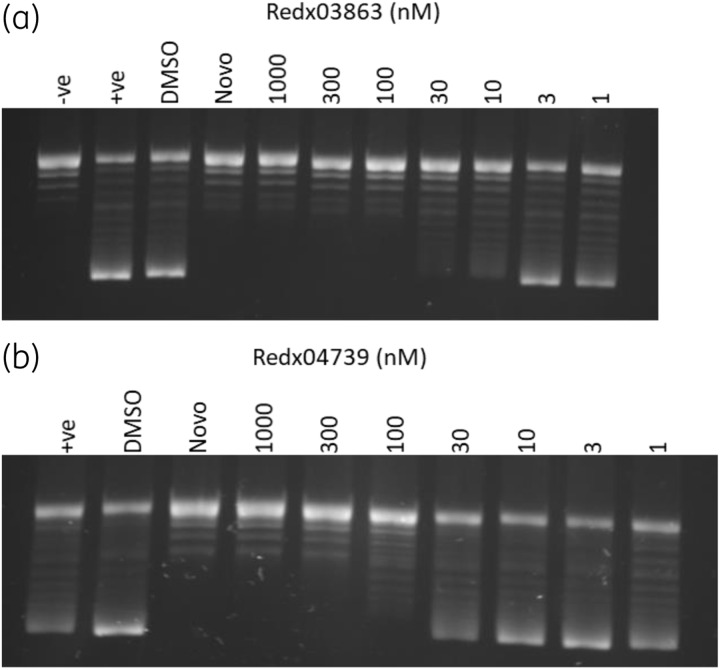
Inhibition of *M. tuberculosis* gyrase supercoiling by Redx03863 and Redx04739. Supercoiling assays using 74 nM of each gyrase subunit in the presence of a range of concentrations of the compounds: (a) Redx03863 and (b) Redx04739 (1 nM–1000 nM in 1% DMSO). Controls: −ve (no enzyme), +ve [gyrase (A_2_B_2_) only, no compound], DMSO (enzyme in 1% DMSO), Novo (10 μM novobiocin in 1% DMSO). Apparent IC_50_ values were determined to be 9 nM for Redx03863 and 30 nM for Redx04739. Enzyme concentration was chosen to give less than 100% supercoiling for better determination of the IC_50_ values.

Redx03863 and Redx04739 compounds were confirmed to inhibit DNA gyrase from *M. tuberculosis* (Figure 2) using a gel-based supercoiling assay; the IC_50_s for *M. tuberculosis* gyrase were determined to be 9.0 nM (Redx03863) and 30 nM (Redx04739). Gyrase can also relax supercoiled DNA in the absence of ATP;^35^ we found that the Redx compounds did not inhibit this reaction. In the presence of FQs, gyrase can cleave DNA due to the cleavage complex being stabilized.^35^ We found that these compounds did not stabilize the cleavage complex (Figure S1). Therefore, we suggest that, consistent with other similar compounds, Redx03863 and Redx04739 could be ATPase inhibitors.^15^^,^^17^^,^^18^

**Figure 3.**
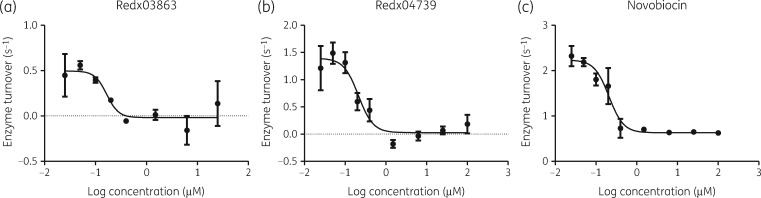
Inhibition of gyrase ATPase activity. Plots of the *M. tuberculosis* gyrase ATPase activity in the presence of Redx03863, Redx04739 and novobiocin. All data are shown as the number of ATP molecules turned over per second by each individual GyrB subunit against the log of the inhibitor concentration. Assays were carried out using the *M. tuberculosis* GyrBA fusion protein (Supplementary data) at 200 nM, in the presence of linear pBR322 DNA (20 μg/mL). IC_50_s were determined to be 0.21 μM (novobiocin), 0.17 μM (Redx03863) and 0.21 μM (Redx04739); note that these are apparent IC_50_s due to the high concentration of enzyme. Error bars represent the range of individual replicates, with a minimum of two repeats.

We directly tested Redx03863 and Redx04739 in gyrase ATPase reactions and found that both compounds inhibit the ATPase reaction of *M. tuberculosis* gyrase with high efficiency, like that of novobiocin (Figure 3), with IC_50_ values of ∼200 nM. As the gyrase concentration used in the ATPase assays was 200 nM it is not feasible to determine IC_50_ values that are lower than this. These data suggest that the binding of Redx03863 and Redx04739 results in reduced ATPase activity, and that their binding site may be close to the ATP-binding site within GyrB.

### Bacterial mutagenesis

Using a plate-based assay at 4× agar MIC of Redx03863, an *M. smegmatis* strain was isolated that was found to have a resistance mutation in the GyrB ATPase region (Gly83Ser; Table S2), which has been previously implicated in resistance to novobiocin.^36^^,^^37^ This mutant strain showed a 16-fold increase in resistance to the parent compound (Redx03863), alongside an 8-fold increase in resistance to Redx04739 and a 32-fold increase in resistance to novobiocin. This cross-resistance suggests that the three compounds bind at a similar site in GyrB.

### Structure of Redx03863-binding pocket

**Figure 4.**
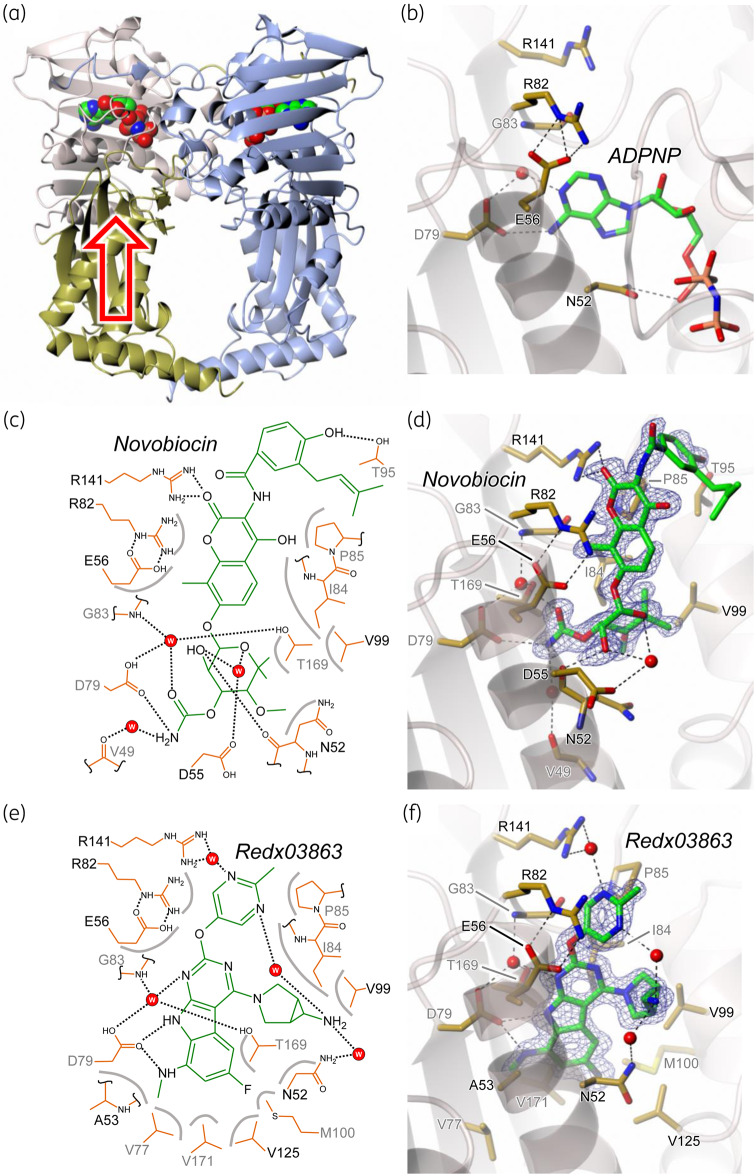
Structures of *M. thermoresistibile* GyrB N-terminal sub-domain complexed with novobiocin and Redx03896. (a) Overview of *M. tuberculosis* GyrB47 homodimer structure (PDB code 3ZKB) in ribbon representation with ADPNP bound (van der Waals spheres). The equivalent of the *M. thermoresistibile* GyrB N-terminal 21 kDa sub-domain is coloured cream with the rest of the subunit in gold. The open red arrow indicates the direction of view for parts (b) to (f). (b) Close-up of the *M. tuberculosis* GyrB ATP-binding site with ADPNP bound in stick representation (green carbons). Also shown in sticks (gold carbons) are the equivalents of residues that were mutated in *M. thermoresistibile* GyrB21 in this study. (c) Two-dimensional schematic showing direct hydrogen bonds and those mediated by a single water molecule between novobiocin and *M. thermoresistibile* GyrB21; van der Waals interactions are indicated by grey arcs. (d) Equivalent of part (c) showing the actual structure with 1.4 Å resolution omit *m*F_obs _− *D*F_calc_ difference electron density contoured at 3.0 σ and the protein backbone traced as a semi-transparent ribbon. Only residues that interact with the ligand (sticks; green carbons), via hydrogen bonds or van der Waals contacts, are included (sticks; yellow carbons). (e) and (f) Representations corresponding to panels (c) and (d), respectively, but for the complex with Redx03896, where the omit *m*F_obs_ − *D*F_calc_ difference electron density is at 1.6 Å resolution and contoured at 3.0 σ.

Redx03863 and Redx04739 impact DNA gyrase activities, suggesting that they are likely to bind at the ATP-binding site of mycobacterial GyrB. Attempts to co-crystallize the Redx compounds with the GyrB ATPase sub-domain from *M. tuberculosis* and *M. smegmatis* were not successful, although we were able to obtain crystals of the *M. smegmatis* protein with novobiocin and solve the structure to 1.6 Å resolution (Figure S2; note that novobiocin has previously been shown to bind to *M. smegmatis* GyrB^38^). We have investigated gyrase from *M. thermoresistibile* and found it to be more stable than the *M. tuberculosis* enzyme (Figure S3). Based on these findings, we successfully co-crystallized a 21 kDa N-terminal sub-domain of *M. thermoresistibile* GyrB (residues 20–254 with residues 103–122 and 214–244 excluded) with novobiocin and Redx03863. This N-terminal sub-domain had been previously used to determine the novobiocin-binding site in gyrase from other organisms.^39–41^ This fragment readily crystallized in the presence of novobiocin, giving diffraction to 1.4 Å, and the structure was solved by molecular replacement (Figure 4), using the structure of the equivalent fragment from *M. smegmatis* with bound novobiocin described above. Superposition of the two mycobacterial novobiocin complexes gave a root mean square deviation (RMSD) value of 0.688 Å, indicating that they are closely similar. Using cross-seeding from crystals of the novobiocin complex, crystals of Redx03863 bound to the 21 kDa fragment were obtained, which diffracted to a resolution of 1.5 Å. The structure was solved using molecular replacement from the novobiocin complex structure (Figure 4). This new crystal structure demonstrated that the binding mode of Redx03863 partially overlaps those of novobiocin and 5′-adenylyl-β,γ-imidodiphosphate (ADPNP; Figures 4 and S4). However, in contrast to novobiocin, Redx03863, is more deeply buried and shows a greater correspondence in atomic positions to ADPNP, most notably in the overlap of the pyrrolopyrimidine and adenine rings (Figure S4).

Residue Arg141 (Arg136 in *E. coli*) makes a key contact to the aminocoumarin ring system of novobiocin;^39–41^ the direct interaction between Arg141 and the aminocoumarin ring system of novobiocin has been crystallographically confirmed, and mutation at this residue leads to resistance to the drug.^39^^,^^42–44^ In the Redx03863 complex, there is only a water-mediated interaction between Arg141 and the ligand (Figure 3). Indeed, when we made site-directed mutations to Ala and Gln at Arg141 in *M. tuberculosis* GyrB, the mutated proteins showed only weak supercoiling activity and were susceptible to inhibition by Redx03863 (Figure S5), i.e. this water-mediated contact does not greatly affect the interaction of Redx03863 with gyrase. When we selected for spontaneous resistance mutations to Redx03863 in *M. smegmatis*, the only mutation that we could isolate was Gly83Ser; no mutations at Arg141 were found, again suggesting it is of minor importance. In relation to Gly83 to Ser, the presence of a side chain at this position would cause steric clashes with the neighbouring structure and most likely displace and/or destabilize the loop bearing residue 83, which also contains several other important ligand-binding residues, namely Arg82, Ile84 and Pro85 (Figure 4e and f). *M. tuberculosis* GyrB containing this mutation (Gly83Ser) showed no activity *in vitro*. The effect on ATP binding is less obvious from the *M. tuberculosis* GyrB47 dimer structure (N-terminal ATPase domain of GyrB),^45^ although it could affect dimer formation and thereby compromise activity. Additionally, based on the crystal structure (Figure 4), we made mutations at Asn52, Glu56, Asp79 and Arg82 (all to Ala) in *M. tuberculosis* GyrB; three of these mutations, or their equivalents, have been found previously to affect ATPase activity.^36^^,^^37^^,^^39^^,^^46^ We found that none of these mutant enzymes showed any supercoiling activity (Figure S6). This suggests that selecting resistance mutations to Redx03863 is difficult without compromising enzyme activity, which is a feature in favour of exploiting these compounds as leads for antimicrobial chemotherapy.

In summary, we have determined the mode of action for Redx03863 and Redx04739, including our proposal for the binding site of Redx03863, as elucidated by X-ray crystallography. Therefore, we conclude that, like other tricyclic pyrrolopyrimidine compounds,^15^^,^^17^ Redx03863 and Redx04739 bind tightly to GyrB within a pocket overlapping with parts of the binding pockets of both novobiocin and ATP, thus preventing ATP turnover and DNA supercoiling from occurring. A large number of compounds that bind at or near the ATP-binding pocket of GyrB have been developed in recent years.^13^^,^^14^^,^^47^^,^^48^ This is largely due to the fact that the crystal structure of the N-terminal sub-domain of *E. coli* GyrB bound to novobiocin has been known since 1996,^41^ enabling the use of computational design and chemical synthesis. Although novobiocin has been used as a clinical antibiotic, it is not currently used. At the time of writing no other GyrB inhibitors that bind to this region are in common clinical use as antibiotics. This is likely due to issues connected with solubility and ease of penetration into bacteria. The fact that GyrB proteins containing mutations in the Redx03863-binding site were largely inactive is potentially a positive feature of these compounds. Taken together with the relatively small size of Redx03863 and Redx04739, their ease of synthesis, and their activity against Gram-negative and Gram-positive bacteria, including *M. tuberculosis*, this suggests that these compounds could have future promise to be further exploited as potential antibiotics.

## Supplementary Material

dkaa286_Supplementary_Data
