# Supplementary material for: Structural and mechanistic analysis of ATPase inhibitors targeting mycobacterial DNA gyrase
Source: J Antimicrob Chemother. 2020 Jul 30;75(10):2835–42. doi: 10.1093/jac/dkaa286 (PMC7556816; doi:10.1093/jac/dkaa286)

**Supplementary data**

**Supplementary Methods**

***Cloning and Purification***

In order to prepare DNA gyrase (GyrA and GyrB) from *M. thermoresistibile*, constructs were designed based on the *M. thermoresistibile* ATCC19527 genomic sequence. The *gyrA* and *gyrB* sequences were codon-optimised using the GeneArt service (Fisher Thermo Scientific). The constructs were cloned into the pET28-MHL expression vector (Addgene) with an N-terminal His-tag and TEV protease sequence via the InFusion cloning system (Takarabio). To make the GyrBA fusion construct, a single amino acid linker (Lys) was added between GyrB and GyrA. After the clones were confirmed as correct via sequencing (SourceBioscience), expression trials were carried out in a range of *E. coli* expression hosts and under a range of conditions. It was determined that the optimum conditions were the expression of the codon-optimised genes in the Rosetta^TM^ 2 (pLysS) *E.* coli strain in LB with induction at OD_600_ of 0.6, by addition of 0.4 mM IPTG at 28°C for 4-5 h.

For large-scale expression, harvested cells were resuspended in 50 mM Tris·HCl pH 7.9, 400 mM NaCl, 20 mM imidazole, 1x cOmplete Mini EDTA-free protease inhibitor tablet (Roche) and stored at -80°C. Purification of the subunits was achieved through a two-column strategy: application to a 5 ml His Trap column (GE) in Lysis Buffer (50 mM Tris·HCl pH 7.9, 400 mM NaCl, 20 mM imidazole) with elution in Lysis Buffer with a gradient from 20 mM to 500 mM imidazole. The semi-purified fractions were dialysed overnight against TEV Cleavage Buffer (50 mM Tris·HCl pH 7.9, 50 mM NaCl, 5 mM DTT) with 1 mg of TEV protease. The flow-through was collected from a second 5 ml His Trap column. In addition, the GyrBA fusion protein was further purified using a Sephacryl S400 size exclusion column in Gel Filtration Buffer (50 mM Tris·HCl pH 7.9, 50 mM NaCl, 5 mM DTT). Pure fractions, analysed via 8% Tris·glycine SDS-PAGE, were collected, concentrated and dialysed against Storage Buffer (50 mM Tris·HCl pH 7.9, 50 mM NaCl, 5 mM DTT, 20% (v/v) glycerol) before concentration, aliquoting and flash freezing to -80°C for storage.

To prepare the *M. tuberculosis* GyrBA fusion protein, a construct was made comprising GyrB fused N-terminally to GyrA, with a single Lys linker. Our original construct (gift of S.J. Hearnshaw and F. Collin) contained the extra 40 amino acids that have been determined not to be biologically relevant.^1^ Both the GyrB and GyrBA constructs were altered by deleting the N-terminal 40 amino acids. The GyrBA coding sequencing was inserted using In-Fusion cloning (Takara Bio) into the pET28-MHL vector at the BseRI site. This introduced an N-terminal His-tag followed by a TEV cleavage sequence before the GyrBA coding sequence. The natural Val start codon was altered to a Met for expression in *E. coli*. It was found that the cells expressed the 170 kDa protein in a variety of different conditions, but optimally in the Rosetta^TM^ 2 (pLysS) cell line, by induction with 0.8 mM IPTG at 28-30°C, for a time greater than 4 hours.

After optimisation of expression on a small scale, the protein was expressed on a large scale (4-8 L). Cells were lysed using an Avestin EmulsiFlex-B15 homogeniser at 40,000 psi. After lysis and a centrifugation step, purification of the clarified lysate was achieved using a His Trap (GE) with elution in Lysis Buffer with an imidazole gradient (to 500 mM). The semi-purified fractions were dialysed overnight against TEV Cleavage Buffer, with 1 mg of TEV protease. The flow-through was collected from a second His Trap column. Protein was further purified using a MonoQ column and a Sephacryl S-400 HR column as required. (Cleavage of the His-tag was not found to affect the activity of the protein, therefore, the TEV protease cleavage step followed by a reverse HisTrap was not always performed.)

**Supplementary Results**

***Expression and purification of DNA gyrase from M. tuberculosis and M. thermoresistibile***

During the course of this work we expressed and purified DNA gyrase from *M. thermoresistibile* (GyrA and GyrB and the GyrBA fusion protein) and also the GyrBA fusion protein from *M. tuberculosis.* The objective of utilising gyrase from a thermo-stable strain, *M. thermoresistibile*,^2^ and constructing GyrBA fusion proteins was to improve the stability of the enzyme for current and future mechanistic and structural work; the approach of using BA fusion proteins has been used previously with other species.^3-8^

***Activities of M. thermoresistibile gyrase***

Having made gyrase proteins from *M. thermoresistibile* (Mth) we wanted to ascertain whether they had the expected enzymatic activities and compare these with those of the *M. tuberculosis* (Mtb) enzyme. We found that all four enzymes, i.e. Mtb and Mth gyrase (A_2_B_2_) and the GyrBA fusion proteins, exhibited ATP-dependent DNA supercoiling activity (Supplementary Figure 3). We found that, in general, the fusion proteins showed increased activity compared to the enzyme made from individual subunits; the supercoiling assays were carried out using conditions described previously,^9^ which we found to be approximately optimal for the Mtb enzyme. We found that the optimal conditions for the Mth enzyme were slightly different (50 mM Tris·HCl (pH 7.5), 200 mM KGlu, 10 mM Mg.OAc, 10 mM DTT, 1 mM ATP, 0.05 mg/mL albumin). In assays with the Mth enzyme, we found that we needed to include a proteinase K step in order to better visualize the DNA on the gel, suggesting that Mth gyrase has a higher affinity for DNA than the Mtb enzyme.

In the absence of ATP, gyrase can relax negatively supercoiled DNA;^10^ we found that all four enzymes (Mtb and Mth gyrase (A_2_B_2_) and GyrBA fusion proteins) were able to carry out this reaction, albeit at a slower rate than the supercoiling reaction. In addition, we found that the enzymes could carry out decatenation using kDNA as a substrate, and also exhibited DNA-dependent ATPase activity (data not shown).

***Temperature dependence of M. tuberculosis and M. thermoresistibile gyrase supercoiling activity***

As discussed above, we expected Mth DNA gyrase to be more resistant to high temperatures than its Mtb counterpart. We therefore carried out supercoiling assays with both enzymes at a range of temperatures. Assays at 37°C suggested that the Mtb and Mth enzymes had similar activities (Supplementary Figure 3). Similar experiments were carried out at a range of temperatures up to 65°C, at which neither enzyme was active. We found that whereas the Mth fusion enzyme still showed some activity up to 60°C, the Mtb fusion enzyme did not show significant activity beyond 47°C (sample data at 55°C are shown in Supplementary Figure 3). Interestingly we found that the A_2_B_2_ enzymes were less thermotolerant in the DNA supercoiling reaction than their fusion counterparts; the maximum temperatures at which activity was seen were ~47°C and ~42°C, for the Mth and Mtb enzymes respectively. This suggests that fusing the subunits also makes a contribution to enzyme stability.

**Supplementary References**

**1**. Karkare S., Brown A. C., Parish T. *et al.* Identification of the likely translational start of Mycobacterium tuberculosis GyrB. *BMC Res Notes* 2013; **6**: 274.

**2**. Edwards T. E., Liao R., Phan I. *et al.* Mycobacterium thermoresistibile as a source of thermostable orthologs of Mycobacterium tuberculosis proteins. *Protein Sci* 2012; **21**: 1093-6.

**3**. Bax B. D., Chan P. F., Eggleston D. S. *et al.* Type IIA topoisomerase inhibition by a new class of antibacterial agents. *Nature* 2010; **466**: 935-40.

**4**. Chen C., Villet R., Jacoby G. A. *et al.* Functions of a GyrBA fusion protein and its interaction with QnrB and quinolones. *Antimicrob Agents Chemother* 2015; **59**: 7124-7.

**5**. Papillon J., Menetret J. F., Batisse C. *et al.* Structural insight into negative DNA supercoiling by DNA gyrase, a bacterial type 2A DNA topoisomerase. *Nucleic Acids Res* 2013; **41**: 7815-27.

**6**. Gubaev A., Klostermeier D. DNA-induced narrowing of the gyrase N-gate coordinates T-segment capture and strand passage. *Proc Natl Acad Sci U S A* 2011; **108**: 14085-90.

**7**. Schoeffler A. J., May A. P., Berger J. M. A domain insertion in Escherichia coli GyrB adopts a novel fold that plays a critical role in gyrase function. *Nucleic Acids Res* 2010; **38**: 7830-44.

**8**. Laponogov I., Pan X. S., Veselkov D. A. *et al.* Structural basis of gate-DNA breakage and resealing by type II topoisomerases. *PLoS ONE* 2010; **5**: e11338.

**9**. Aubry A., Fisher L. M., Jarlier V. *et al.* First functional characterization of a singly expressed bacterial type II topoisomerase: the enzyme from Mycobacterium tuberculosis. *Biochem Biophys Res Commun* 2006; **348**: 158-65.

**10**. Gellert M., Mizuuchi K., O'Dea M. H. *et al.* Nalidixic acid resistance: a second genetic character involved in DNA gyrase activity. *Proc Natl Acad Sci U S A* 1977; **74**: 4772-6.

**11**. Karkare S., Chung T. T., Collin F. *et al.* The naphthoquinone diospyrin is an inhibitor of DNA gyrase with a novel mechanism of action. *J Biol Chem* 2013; **288**: 5149-56.

**Supplementary Figure Legends**

**Figure S1**. Redx compounds do not stabilise the DNA cleavage complex with *M. tuberculosis* gyrase. DNA cleavage assays were carried out as previously described;^11^ these are essentially the same as supercoiling assays, except that the reactions are terminated by the addition of SDS and proteinase K, and incubated for a further 30 mins at 37°C. Samples contained 20 nM gyrase and 1% DMSO, except where indicated, and Moxifloxacin, Redx03863 or Redx04739, as indicated. Agarose gels (1%) were run in the presence of 0.5 µg/mL ethidium bromide. – indicates no gyrase; + and all other lanes contain gyrase. nc = nicked circular DNA; l = linear; cc = closed circular.

**Figure S2.** Structure of *M. smegmatis* GyrB N-terminal sub-domain complexed with novobiocin (sticks; green carbons) with associated 1.6-Å resolution omit *m*Fobs-*D*Fcalc difference electron density contoured at 4.0 σ. The protein backbone is traced as a semi-transparent ribbon and residues that interact with the ligand via hydrogen bonds (direct or through a single water molecule) or van der Waals contacts are also shown (sticks; yellow carbons).

**Figure S3.** Supercoiling assays of the GyrBA fusion proteins. Supercoiling time courses for GyrBA fusion proteins from *M. tuberculosis* (Mtb: A, C) and *M. thermoresistibile* (Mth: B, D) at 37°C (A, B) and 55°C (C, D), taken at the time points indicated under identical conditions.

**Figure S4.** Structures of *M. thermoresistibile* GyrB N-terminal sub-domain complexed with novobiocin and Redx03896. (A) – (C) Pairwise superpositions of the ligands from the novobiocin and Redx03896 complexes of *M. thermoresistibile* GyrB21 with the ADPNP complex of *M. tuberculosis* GyrB47, where the ligands are shown in stick representation (see colour keys) and the backbone trace depicted as a semi-transparent ribbon. (D) Illustration of the fit of Redx03896 in the ATP pocket, with the protein depicted as a molecular surface and the ligand as van der Waals spheres. The direction of view corresponds to that indicated by the open red arrow in part (C).

**Figure S5**. Inhibition of *M. tuberculosis* gyrase supercoiling, WT and mutants, by Redx03863 and Redx04739. WT gyrase (30-min incubation), Arg141Glu (60 min), and Arg141Glu (75 min) GyrB mutants (Note that longer times were needed for the mutants due to low activity.) Supercoiling assays using 78 nM of each gyrase subunit in the presence of a range of concentrations of the compounds (10-1000 nM in 1% DMSO. -ve (no enzyme), +ve (gyrase (A_2_B_2_) only, no compound), DMSO (enzyme in 1% DMSO), Novo (10 µM novobiocin in 1% DMSO). (Enzyme concentration was chosen to give less than 100% supercoiling for better determination of the IC_50_ values; note that at this novobiocin concentration the WT was completely inhibited and the mutants were at least partially inhibited.)

**Figure S6** GyrB mutants showing no supercoiling activity. Supercoiling assay with 5 mutants made in the GyrB subunit of Mtb DNA gyrase, suggested by X-ray crystallography to be in the binding site of Redx03863. Activity was tested with equal concentrations of GyrA and GyrB mutants (as indicated) for 30 mins at 37°C. A mutant was declared inactive if it displayed no apparent supercoiling activity at 200 nM concentration under these conditions.

**Supplementary Tables**

**Table S1.** Primers used for DNA amplification and sequencing of the *M. smegmatis* *gyrA* and *gyrB* genes.

| *M. smegmatis* *gyrB* F | ATAGGTGGAAACGCGGCTAC |
| --- | --- |
| *M. smegmatis gyrB* F1 | GCTGGAGGCCACGGTCCTG |
| *M. smegmatis gyrB* F2 | TCGAGATCGCGATGCAGTGGA |
| *M. smegmatis gyrB* F3 | CGACATCGGTGGGTTGCCG |
| *M. smegmatis gyrB* R | GGCAGCGTCGTATCAGTCAT |
| *M. smegmatis gyrA* F | ACGTTCGCTTCCTGGATGTT |
| *M. smegmatis gyrA* F1 | TGGCGATGGAGATGTTGCGTG |
| *M. smegmatis gyrA* F2 | CAGGTGCGCGACGGCAAG |
| *M. smegmatis gyrA* F3 | CCTCGAGCGGCAGAAGATCG |
| *M. smegmatis gyrA* F4 | AACCTCCTGGCCTTCCAGCC |
| *M. smegmatis gyrA* R | CCTACAGCTCCTTAGCTCGG |

**Table S2.** MIC_100_ values for mutants raised against Redx03863.

|  | Redx03863 (µg/ml) | Redx04739 (µg/ml) | Moxifloxacin (µg/ml) | Novobiocin (µg/ml) | Isoniazid (µg/ml) |
| --- | --- | --- | --- | --- | --- |
| *M. smegmatis* ATCC19420 (WT) | 0.016 | 0.5 | 0.125 | 2 | 32 |
| *M. smegmatis* 4x03863 Mutant 1 | 0.25 | 4 | <0.125 | 4 | 32 |
| ***M. smegmatis* 4x03863 Mutant 2** | **0.25** | **4** | **<0.125** | **64** | **32** |
| *M. smegmatis* 4x03863 Mutant 3 | 0.125 | 4 | <0.125 | 8 | 16 |
| *M. smegmatis* 4x03863 Mutant 4 | 0.25 | 2 | <0.125 | 4 | 32 |
| *E. coli* ATCC25922 (WT) | 0.016 | 8 | 0.016 | 64 | - |
| *E. coli* 2x03863 Mutant | 32 | >64 | 0.016 | 0.5 | - |

*M. smegmatis* mutants raised against Redx03863 at 4x agar MIC_100_ and *E. coli* mutants raised against Redx03863 at 2x agar MIC_100_. A concentration range of drugs of 0.125-64 µg/ml was tested. *M. smegmatis* Mutant 2, highlighted in bold, was found to have a mutation in *gyrB* leading to a Gly83Ser mutation in the GyrB protein

**Supplementary Figures**

**Figure S1**


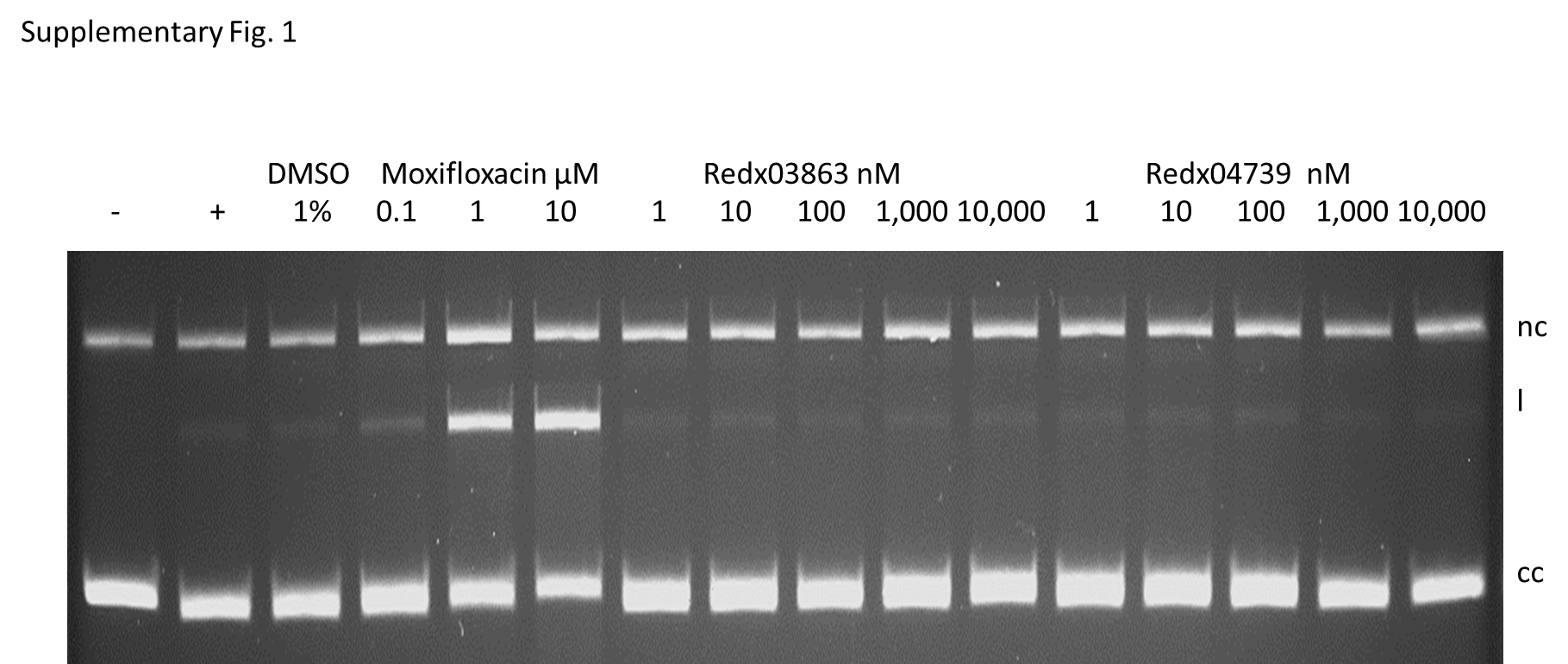


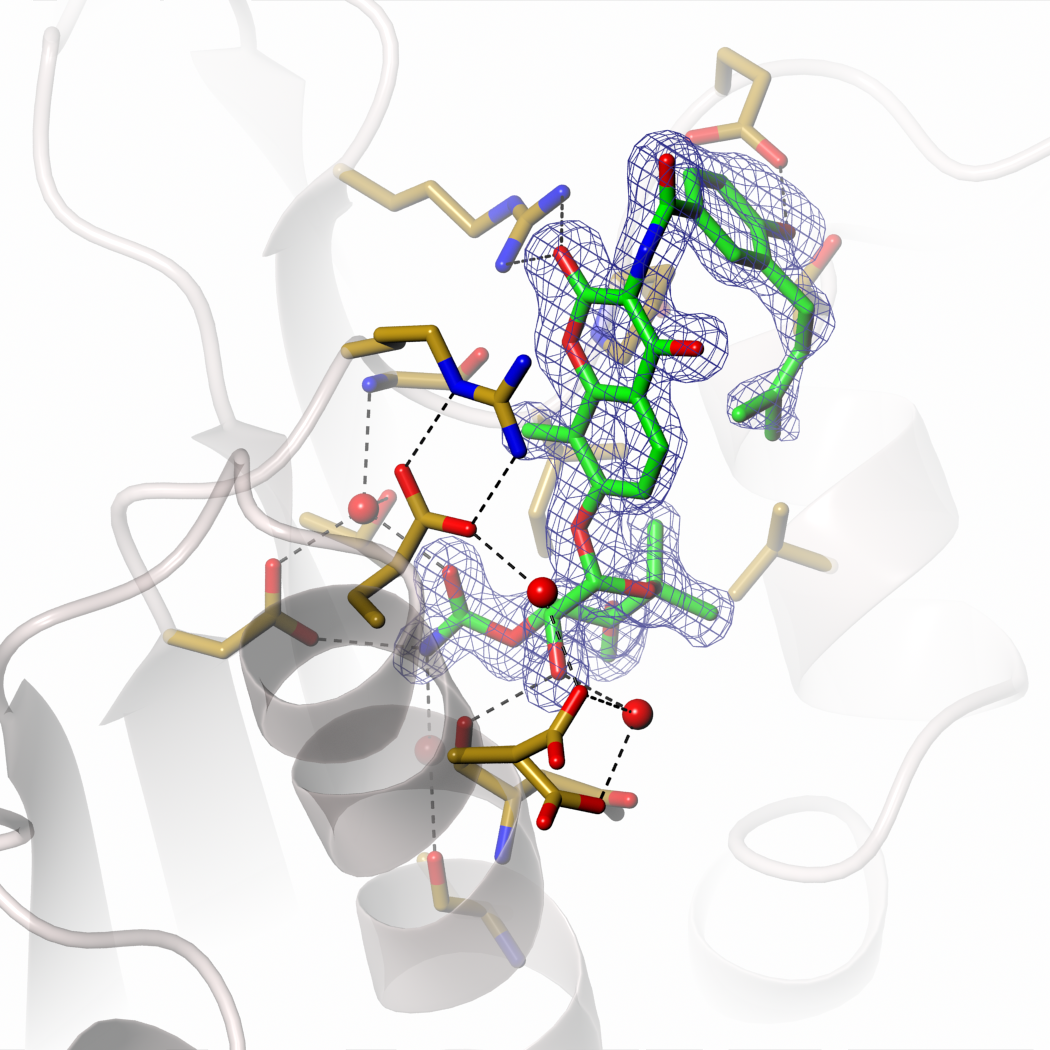


R141

R82

E56

T169

D79

V49

N52

V99

I84

P85

G83

T95

*Novobiocin*

E87

D55

**Figure S2**

**Figure S3**


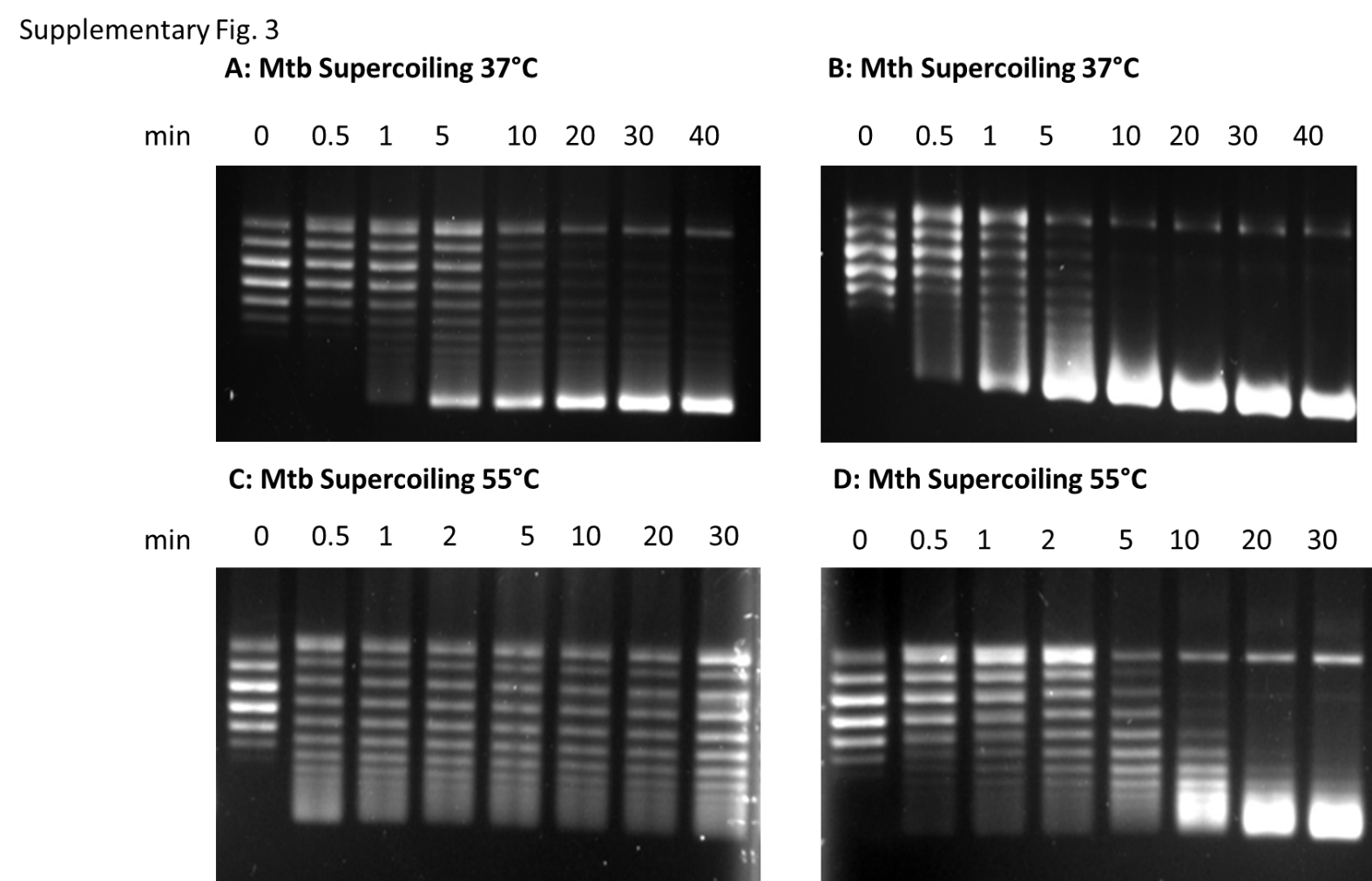


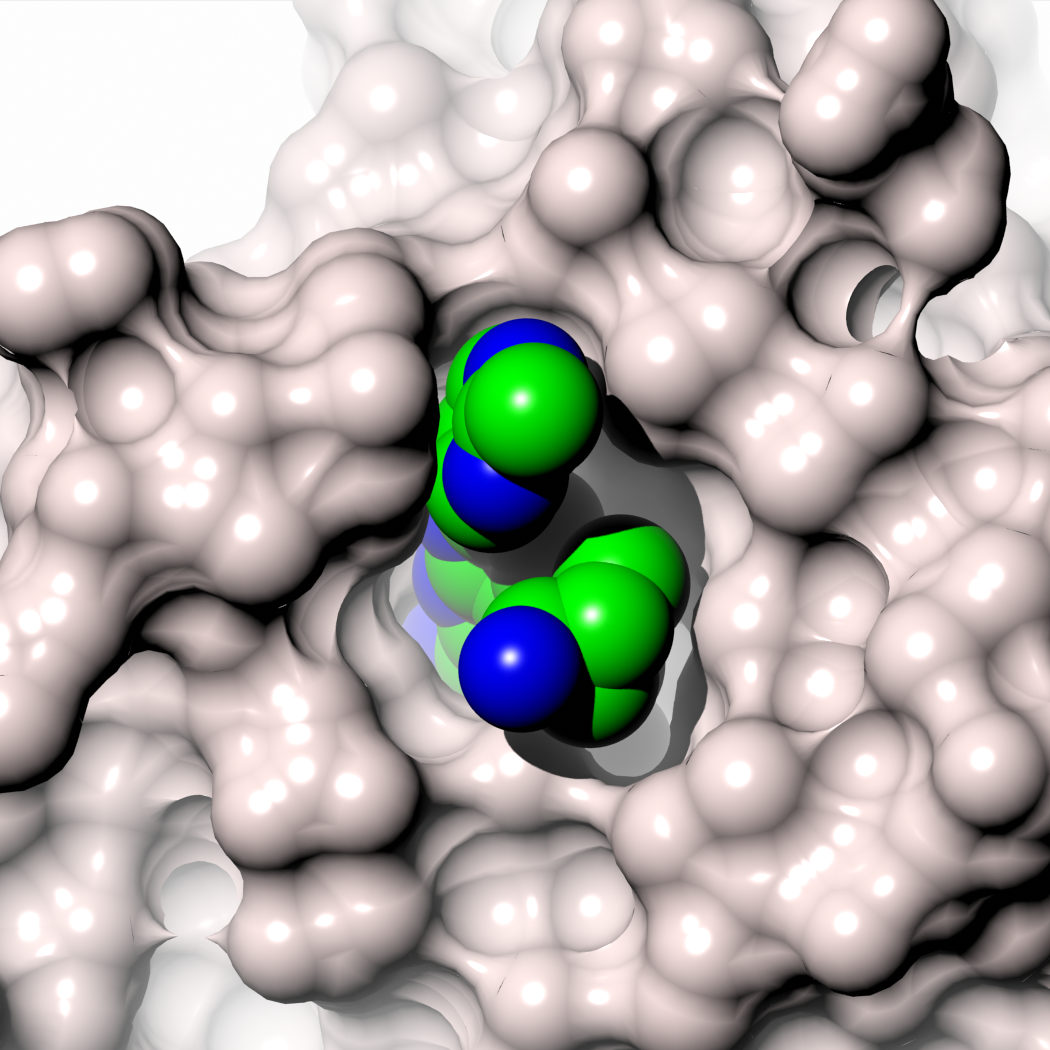

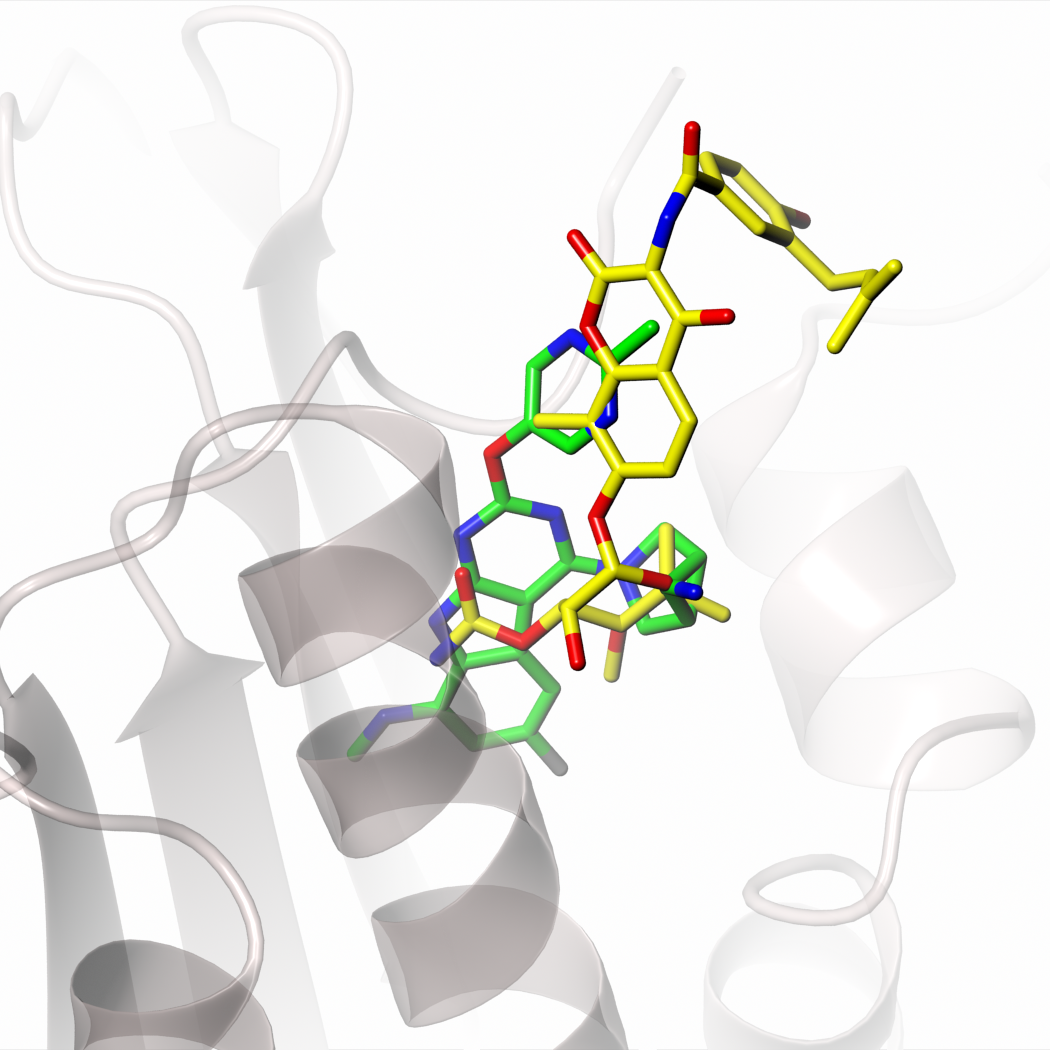


*Novobiocin*

*Redx03863*


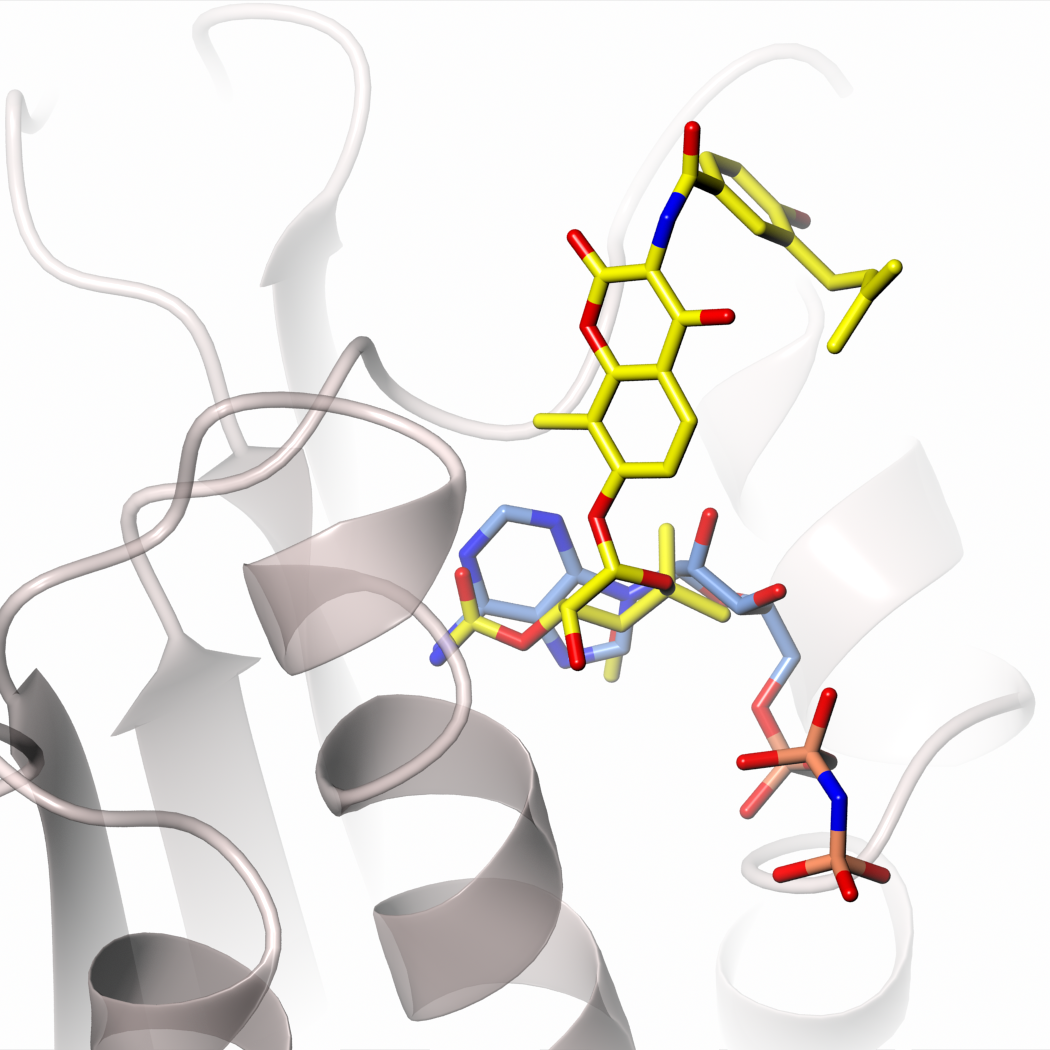


*ADPNP*

*Novobiocin*


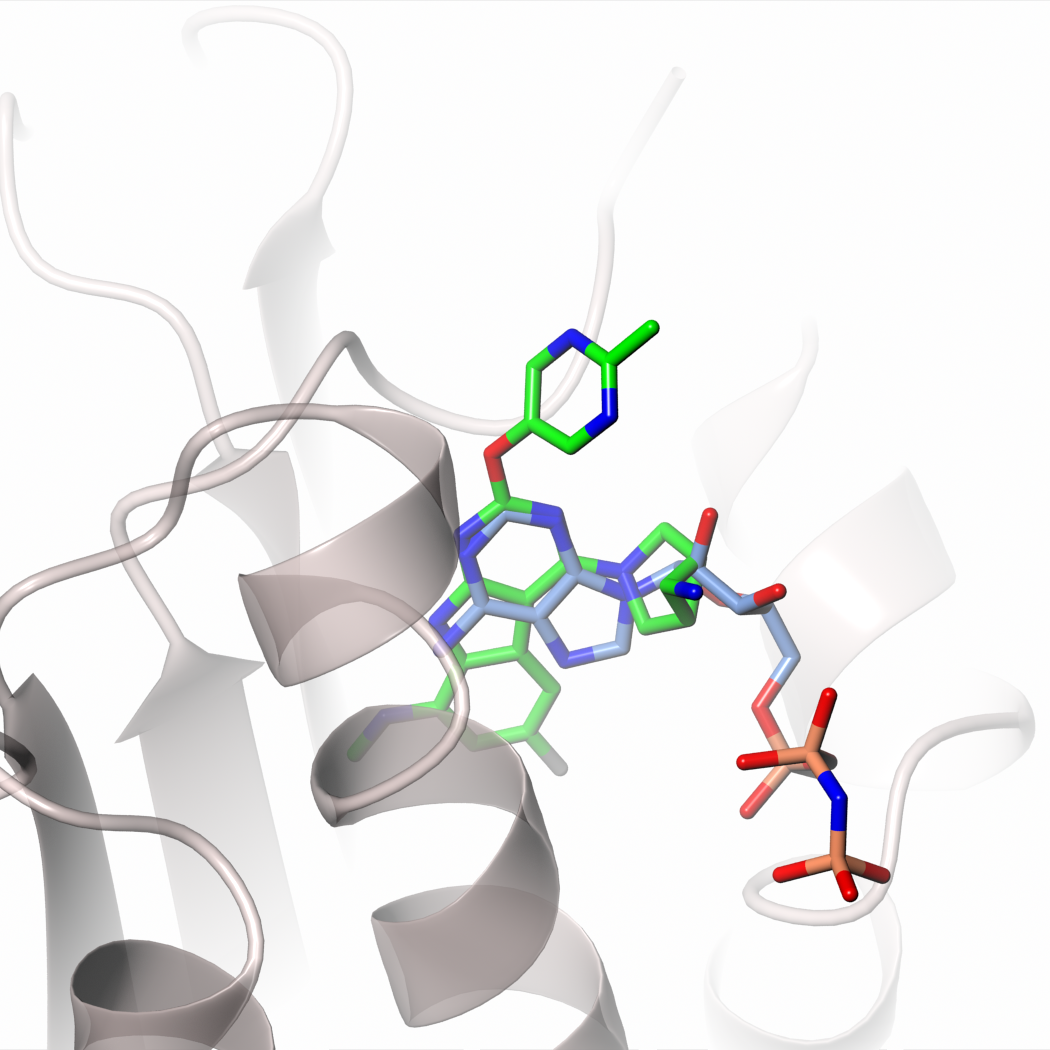


*Redx03863*

*ADPNP*

A

B

C

D

**Figure S4**

**Figure S5**


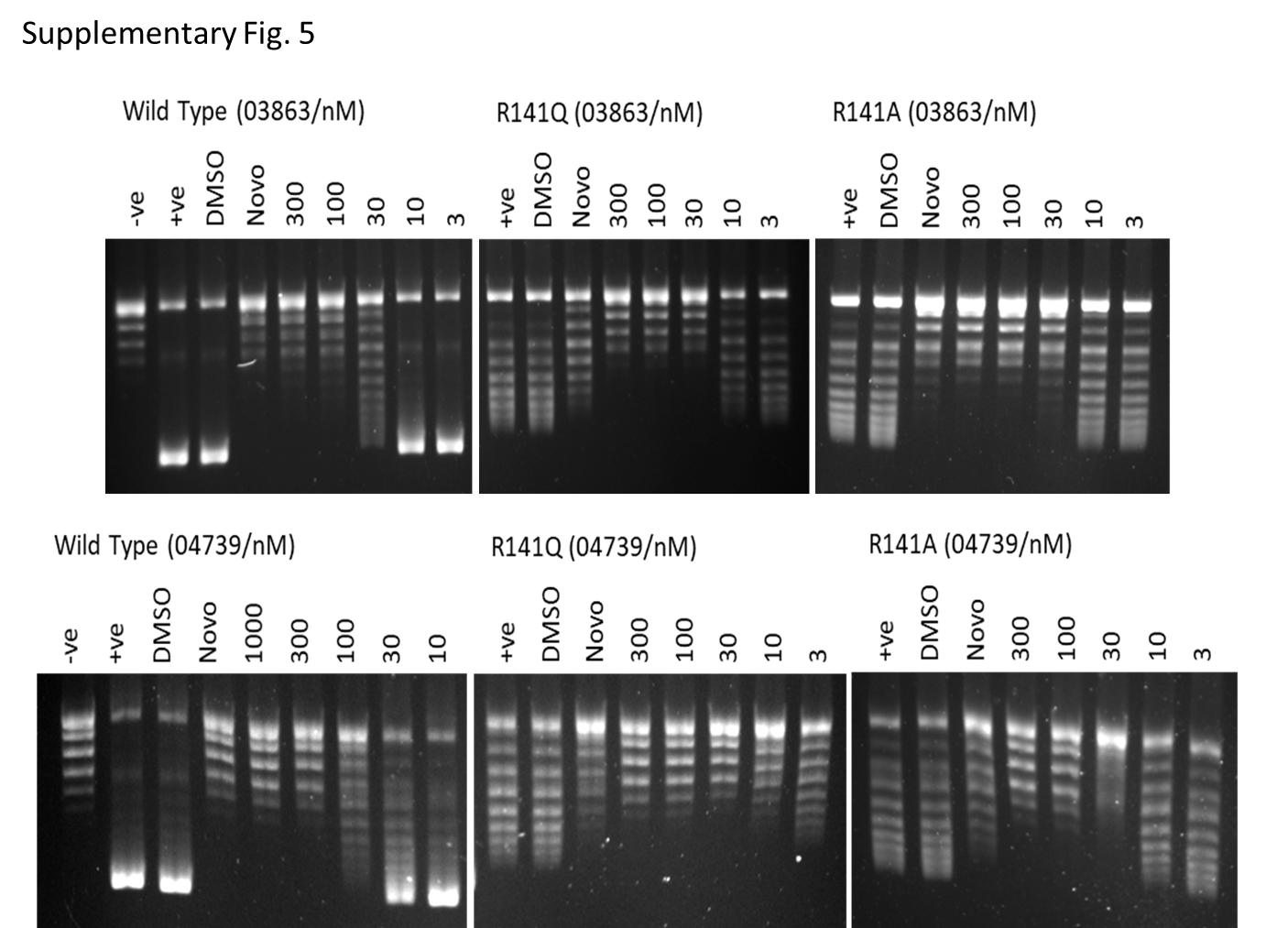


**Figure S6**


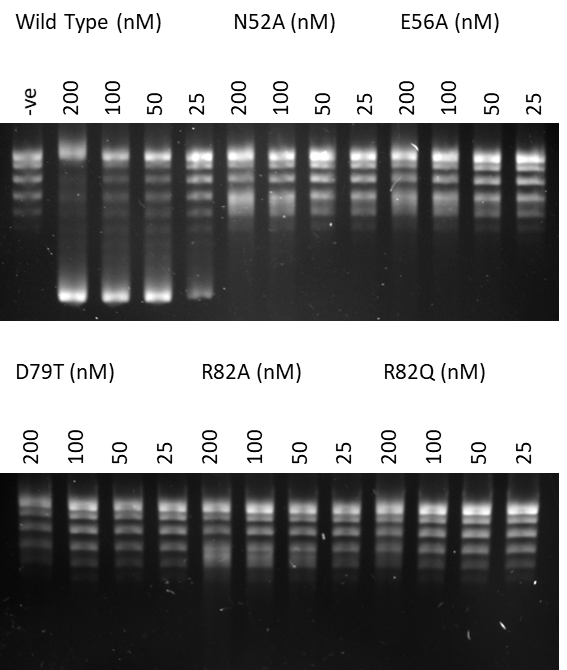

Supplement: dkaa286_Supplementary_Data [file dkaa286_supplementary_data.docx]
